# The complete chloroplast genome sequence and phylogenetic analysis of the medicinal plant *Lysimachia hemsleyana*

**DOI:** 10.1080/23802359.2019.1688115

**Published:** 2019-11-08

**Authors:** Zhiqi Ying, Qirui Wang, Shuisheng Yu, Guanghui Liao, Yuqing Ge, Rubin Cheng

**Affiliations:** a College of Pharmaceutical Science, Zhejiang Chinese Medical University, Hangzhou, China;; b The Administration Bureau of Zhejiang, Jiulongshan National Nature Reserve, Suichang, China;; c The First Affiliated Hospital of Zhejiang, Chinese Medical University, Hangzhou, China

**Keywords:** Lysimachia hemsleyana, chloroplast genome, phylogenetic analysis, medicinal plant

## Abstract

*Lysimachia hemsleyana Maxim.* is an important medical plant in the Family Primulaceae. In this study, we determined the complete chloroplast genome of *L. hemsleyana.* It is 155,618 bp in length, containing a large single copy (LSC) region of 85,615 bp, a small single copy (SSC) region of 17,861 bp, which were separated by a pair of inverted repeat (IR) regions of 26,071bp. The complete chloroplast genome of *L. hemsleyana* encoded a total of 134 genes, including 89 protein-coding genes with the pseudogene of ycf1, 8 ribosomal RNA genes and 37 transfer RNA genes. Phylogenetic analysis revealed that *L. hemsleyana* was most closely related to the Korea endemic plant *Lysimachia coreana* with high bootstrap support value. This work provides basic molecular information that would be useful for further investigation on conservation genetics and evolutionary relationships of *L. hemsleyana*.

*Lysimachia hemsleyana Maxim.* is an important medical plant belonging to the genus Lysimachia in family Primulaceae. This plant wildly grows in forests and streamside in valleys of China and is widely used in traditional Chinese medicine to treat chronic liver and kidney diseases (Maximowicz 1996). Furthermore, the extraction of *L. hemsleyana* has shown immunoregulation effects in mice, indicating a potential for the treatment of inflammatory pathological process and some autoimmune diseases (Zhang et al. 2002). As the species diversity and similar morphological features in the genus *Lysimachia*, the misidentified crude herbs would be used as adulterations for *L. hemsleyana* and could cause serious health problems. The complete chloroplast genome sequence of the medical plant *L. hemsleyana* could provide the basic molecular data and contribute to the development of molecular identification and further conservation strategy (Shen et al. 2017).

Here, we characterized the complete chloroplast genome sequence of *L. hemsleyana* and investigated the phylogenetic relationship of genus Lysimachia among the family Primulaceae. Total genomic DNA was extracted from fresh leaves of *L. hemsleyana* sample DXH-13 collecting from Fuyang area in Zhejiang Province of China (30°05’2.4”N 119°53’20.4”E). The specimen was deposited in the collection center of Zhejiang Chinese Medical University. The total genomic DNA was sequenced using the Illumina Hiseq Platform (Illumina, San Diego, CA, USA). The complete chloroplast genome of *L. hemsleyana* was assembled from the extracted cp-like reads by MitoZ with the complete chloroplast genome of *Lysimachia coreana* as the reference (Meng et al. 2019). We annotated the assembled chloroplast genome via CPGAVAS and further confirmed it by BLAST (Liu et al. 2012). The final complete cp genome of *L. hemsleyana* was submitted to GenBank with the accession number of MN519195.

The complete *L. hemsleyana* chloroplast genome is a circular DNA molecule with the length of 155,618 bp, containing a pair of IR regions of 26,071 bp that separate a LSC region of 85,615 bp from a SSC region of 17,861bp. The overall GC content of the complete chloroplast genome is 36.9%, and the corresponding values for LSC, SSC, and IR regions are 34.7%, 30.3%, and 42.7%, respectively. In addition, a total of 134 genes are identified, containing 89 protein-coding genes which include a pseudogene of ycf1, 8 rRNA genes, and 37 tRNA genes. Moreover, the complete cp genome of *L. hemsleyana* includes 20 duplicated genes in the IR and 51.5% protein-coding sequences.

Phylogenetic analysis was performed using the complete cp genomes of *L. hemsleyana* with those of 15 species in family *Primulaceae* by maximum-likelihood (ML) method of MEGA 7.0 (Kumar et al. 2016). The complete chloroplast genome sequences were firstly aligned using MAFFT version 7 (Katoh and Standley 2013). The result indicated that *L. hemsleyana* and Korea endemic plant *Lysimachia coreana* clustered together with high statistical support, indicating a close genetic relationship (Son and Park 2016). Furthermore, the genuses *Lysimachia* and *Ardisia* consisted into a monophyletic group with high bootstrap values, which is sister to the clades combined by *Primula* and *Androsace* species (Figure 1). This study provides basic molecular data of *L. hemsleyana*, which was important for the development of specific DNA barcodes and understanding of evolutionary history and conservation strategy in Family Primulaceae.

**Figure 1.**
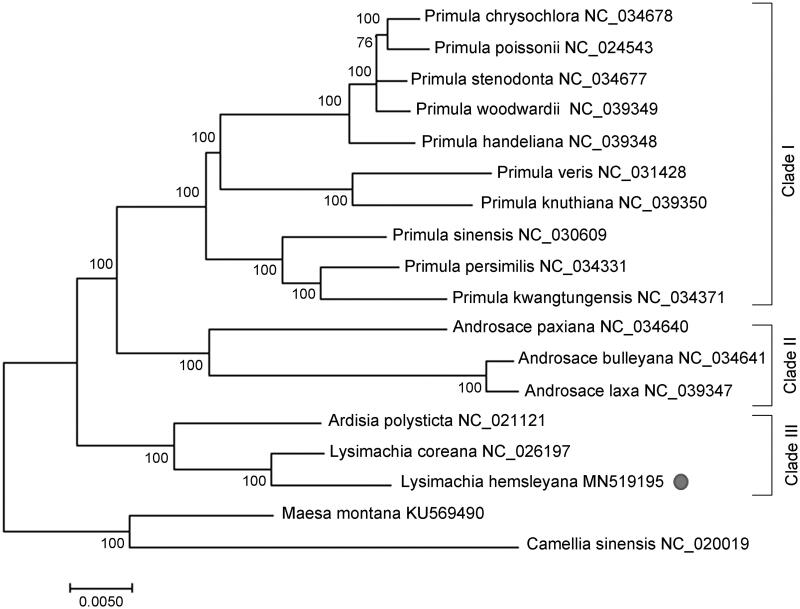
ML phylogenetic tree of *Lysimachia hemsleyana* and other representative Primulaceae plants based on the complete chloroplast genome sequences. Numbers on the nodes are bootstrap values from 100 replicates. *Maesa montana* and *Camellia sinensis* were selected as the outgroup. The GenBank accession numbers were listed following the species name.
